# Effect of simplified exercise program on quality of life, biomarkers, pain and muscle strength of individuals with knee osteoarthritis

**DOI:** 10.31744/einstein_journal/2025AO1192

**Published:** 2025-11-13

**Authors:** Rosana Ravagnani Campedelli, Mariana Rosada de Souza Jardim, Eliane Antonioli, Felipe Bruno Dias de Oliveira, Sudha Agarwal, Mario Ferretti

**Affiliations:** 1 Hospital Israelita Albert Einstein São Paulo SP Brazil Hospital Israelita Albert Einstein, São Paulo, SP, Brazil.; 2 Ohio State University Department of Orthopaedics Division of Oral Biology Columbus Ohio United States Division of Oral Biology and Department of Orthopaedics, Ohio State University, Columbus, Ohio, United States.

**Keywords:** Osteoarthritis, knee, Muscle strength, Biomarkers, Exercise therapy, Pain measurement, Quality of life

## Abstract

**Objective:**

To evaluate a simplified exercise program for patients with knee osteoarthritis in terms of pain, functionality, muscle strength, and quality of life. The correlation of serum levels of molecules and cytokines was evaluated after the exercise program.

**Methods:**

Overall, 47 patients with knee osteoarthritis participated in this single-arm interventional study. Outcomes were assessed using the Western Ontario and McMaster Universities Osteoarthritis Index, Quality of Life, Visual Analog Scale, Timed Up and Go functional test, and muscle strength assessment. Serum levels of molecules and cytokines were analyzed using enzyme-linked immunosorbent assays.

**Results:**

The program resulted in improvements in pain and physical function (0.21 points; 95%CI=-0.26; −0.17), Visual Analog Scale (1.50; 95%CI=-1.90; −1.10), EuroQol-5 Dimension 3-Level (0.16; 95%CI=0.100; 0.227), and Timed Up and Go test (0.40; 95%CI=-0.57; −0.23). Significant differences were noted in the isokinetic knee flexion peak torque (0.16; 95%CI=0.10; 0.22), isometric knee extension (0.31; 95%CI=0.20; 0.43), and flexion (0.18; 95%CI=0.12; 0.23) peak torques for osteoarthritis. However, there was no significant difference in serum biomarker levels after the program, and there was no correlation between serum biomarker levels and clinical improvement outcomes.

**Conclusion:**

The simplified exercise program for patients with knee osteoarthritis patients effectively improved pain, functionality, muscle strength, and quality of life. However, biomarkers do not provide evidence of clinical improvement.

**ClinicalTrials.gov Identifier:**

NCT02964624.

## Highlights

The exercise program reduced the WOMAC score by 50%, VAS score by 53%, and improved isokinetic strength.

There were no significant changes in levels of serum COMP, MMPs, or pro-inflammatory cytokines.

Biomarker levels did not correlate with clinical improvement outcomes.

This exercise program is feasible for knee osteoarthritis management in primary care.

## In Brief

A simplified, low-cost, 8-week resistance exercise program improved pain, function, quality of life, and muscle strength in patients with knee osteoarthritis. However, no significant changes were observed in the inflammatory or cartilage-related serum biomarkers.

## Graphical abstract

**Figure:**
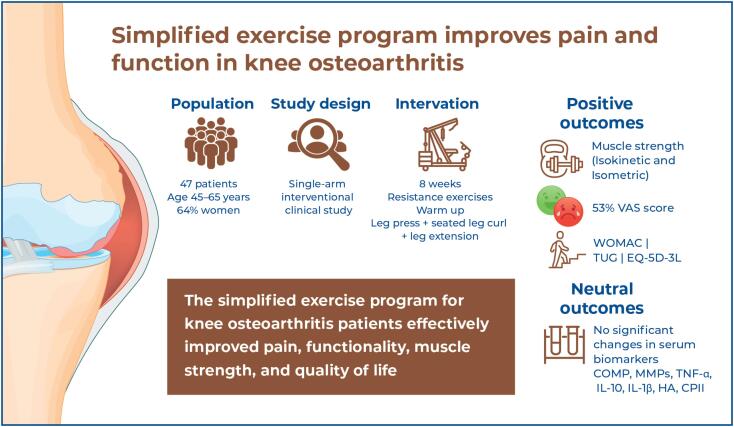

## INTRODUCTION

Osteoarthritis (OA) is the most common type of arthritis worldwide and is associated with morbidity. The prevalence of OA is significantly increasing due to an aging population. Joint pain and stiffness, reduced occupational and social participation, and poor quality of life are common in patients with OA.^( 1 )^ Conservative modalities for OA treatment involve a comprehensive approach, including biomechanical interventions, intra-articular corticosteroids, structured exercise programs, self-management, pain education, and weight management. Additionally, research supports a wide range of appropriate exercise options, including walking, strength training, neuromuscular training, and aquatic exercise. These interventions have demonstrated significant benefits in terms of short-term symptom relief, long-term management of knee pain, and functional improvement.^( 2 )^

Although there is evidence that various exercises have beneficial effects on pain, joint function, and quality of life in patients with OA, an effective exercise prescription has yet to be established. Strength training exercise is essential for knee OA and is recommended by international guidelines, although the ideal exercise volume ("dose") has not been quantified.^( 3 )^ As knee OA is a public health problem, a less expensive approach is desirable. Simple conservative treatment with exercises for the three large lower limb muscle groups may improve patients’ pain and functionality.

Preventive strategies for OA development and follow-up depend on a better understanding of its associated molecular mechanisms. Nevertheless, the relationship between circulating levels of biomarkers related to the pathogenesis and progression of OA remains unclear.^( 4 - 6 )^ Mechanical unloading and overloading, as seen in disuse and overuse, lead to the upregulation of several proinflammatory molecules and enhance tissue degradation,^( 7 , 8 )^ whereas dynamic moderate mechanical loading exerts anti-inflammatory and anti-catabolic effects on articular cartilage through the suppression of inflammation mediators.^( 9 - 11 )^

Regular physical activity reduces levels of inflammatory biomarkers such as pro-inflammatory cytokines (TNF-ALPHA and IL-1 BETA) and catabolic proteins (matrix metalloproteinases) that contribute to cartilage degradation. In contrast, exercise can increase the production of anti-inflammatory and anabolic biomarkers, such as insulin-like growth factor 1 and low-level C-reactive protein, which promote the synthesis of extracellular matrix and cartilage repair.^( 12 , 13 )^ However, the lack of robust biomarkers to evaluate the effectiveness of physical therapy/exercise represents a critical gap in biotechnology, obliterating progress in the optimal application of these therapies.

## OBJECTIVE

This study aimed to evaluate a simplified exercise program for knee osteoarthritis in terms of pain, functionality, muscle strength, and quality of life as well as to correlate serum levels of molecules and cytokines with improvements after the program.

## METHODS

### Study design

This was a single-arm interventional study involving participants recruited from the community through social media (from January 2017 to January 2019) and conducted at the *Centro de Reabilitação do Hospital* in São Paulo, Brazil. The study was approved by the local research ethics committee of the *Hospital Israelita Albert Einstein* (CAAE: 55053616.1.0000.0071; # 3.237.059).

The inclusion criteria were as follows: (1) age between 45 and 65 years, (2) clinical presence of knee OA according to the American College of Rheumatology Clinical Criteria, and (3) Kellgren and Lawrence (KL) radiographic grades 2 and 3 OA (mild to moderate OA).^( 14 )^

The exclusion criteria were as follows: (1) history of major musculoskeletal impairments in the lower limbs or spine; (2) heart conditions or cancer; (3) body mass index (BMI) >35 kg/m^2^; (4) history or scheduled for arthroplasty or osteotomy in the lower limb; (5) corticosteroid injections within the previous 6 months; (6) missing more than two or two consecutive programs; (7) engaging in physical activity more than twice a week; and (8) presence of symptomatic patellofemoral OA.

In the presence of bilateral OA, the study protocol was applied to the knee with the worst symptoms, according to the participant. All participants received verbal and written information regarding the study and signed an informed consent form.

### Simplified exercise program

The program consisted of three exercise sessions per week for 8 weeks and was administered by a physiotherapist. Each session consisted of the following: 1) initial pain assessment (if pain was greater than 4, an analgesic protocol containing ultrasound, laser, or electroanalgesia would be applied), 2) warming up for 10 minutes on a cycle ergometer for 5 minutes at free cadence, with intensity adjusted to 90% of the intensity obtained during the incremental test, and 3) four series (8 to 12 repetitions) of 3 resistance exercises (leg press, seated leg curl, and leg extension machine performed with both lower limbs concomitantly) at 70% of the test load of one-repetition maximum (1RM). At the end of each session, pain was reassessed, and stretching exercises for the thigh muscles were performed.

### Primary outcome

The Western Ontario and McMaster Universities Osteoarthritis (WOMAC) index was used to evaluate the participants’ pain severity and physical function before and after the 8-week program.^( 15 )^

### Secondary outcome

1. The timed up and go (TUG) test was used to assess functional mobility. TUG involved timing a participant as they rose from a chair, walked 3 meters, turned around, walked back, and sat down.^( 16 )^
2. The EuroQol 5-Dimension 3-Level (EQ-5D-3L) quality-of-life scale was administered to assess the participants’ quality of life. This instrument captures a participants’ perceptions across the following five dimensions: mobility, self-care, usual activities, pain/discomfort, and anxiety/depression. It is a validated tool frequently used in health-related quality-of-life assessments.^( 17 )^
3. The pain score was measured through the participants’ answers to a question related to the pain score on the Visual Analog Scale (VAS) from 0 to 10, with 0 indicating no pain and 10 indicating the worst pain imaginable.^( 18 )^
4. Isokinetic Strength Assessment: Isokinetic strength was measured using a Biodex dynamometer (Biodex, Shirley, NY, USA). Peak torque (PT) (Newton meters/kg) was obtained during isokinetic knee extension and flexion and maximal voluntary contraction (MVC). A dynamometer chair and lever arm were attached to the participants. Isometric force was measured using the isometric PT during MVC in isometric knee extension and flexion. For extensor and flexor MVC, the angles between the thigh and leg were 75° (0°=total extension) and 40°, respectively. After familiarization with the protocol, two 5-second MVCs were performed with a 3-minute interval between attempts, and the highest value between them was considered the isometric extension and flexion PT, respectively.^( 19 )^
5. Strength Ratio: Isokinetic strength was measured by the concentric isokinetic PT during MVC at 60°.s^-1^ of the knee flexors, extensors, and isokinetic work. The angular range of motion was 90° (0°=total extension). After familiarization with the protocol, five consecutive MVCs of knee flexion and extension were performed. The highest value among the attempts was considered the concentric isokinetic knee flexor and extensor PT and was used to determine the ratio of strength balance between the knee flexors and extensors (F/E). The data obtained were normalized to body weight and variability in relation to the anthropometric differences between men and women.^( 20 )^
6. Repetition Maximum (1RM) test: The test commenced with a warm up of 5-10 repetitions with moderate load estimation (40%-60% of 1RM). After a 1-minute rest, 3–5 repetitions were performed with a higher load (60%-80% of 1RM) followed by a 5-minute rest. Subsequently, an estimated 1RM attempt was performed. If the patient failed to lift the load within two attempts, the test was halted; otherwise, the load was increased after a 5-minute rest, and the test was repeated. Only five attempts were made per day to maintain the participants’ confidence.^( 21 )^
7. Incremental Test: After warming up for 5 minutes on a cyclergometer under a comfortable cadence, the intensity was increased every 3 minutes until a voluntary Borg scale score of 11–13 was achieved.^( 22 )^
8. Biomarker assessments: Serum samples were obtained via venous blood collection from the antecubital vein before (Pre), after the 12th session (Int), and at the end (Post) of the simplified exercise program. The samples were centrifuged, aliquoted, and stored at −80º. Human magnetic Luminex assay and ELISA were employed to detect inflammatory markers, collagen degradation, and cartilage synthesis.

### Inflammatory markers

The following nine molecules and cytokines associated with inflammation were analyzed: TNF-ALPHA, IL-6, IL-8/CXCL8, IL-10, IL-1 BETA/IL-1F2, IL-17E/IL-25, CL3/MIP-1 ALPHA, LEPTIN, and IL-18 (cat # LXSAHM 9 Plex, R&D Systems).

### Cartilage degradation

The following biomarkers associated with cartilage degradation were quantified: matrix metalloproteinases (MMP-1, MMP-3, and MMP-13;cat #LMPM, 3 Plex; R&D Systems), C2C (a neoepitope generated by collagenase-mediated cleavage of type II collagen; Ibex Pharmaceuticals Inc., cat. #60-1001-001), CS846 (an epitope located on chondroitin sulfate chains of the cartilage proteoglycan aggrecan; cat. #60-1004, Ibex Pharmaceuticals Inc.), COMP (cartilage oligomeric matrix protein; cat. #DCMP0, R&D Systems), HA (hyaluronic acid, cat. #DHYAL0, R&D Systems,), and HMGB1 (high mobility group box1 protein; cat. #SEA399Hu, Cloud-Corp).^( 23 )^

### Collagen synthesis

The biomarkers related to collagens synthesis analyzed were: CPII (type II collagen carboxypropeptide cat. #60-1003-001, Ibex Pharmaceuticals Inc.), and Aggrecan (cat. LXSAHM-01 Plex, R&D Systems).

### Statistical analysis

The sample size was based on the the minimal clinically important difference (MCID) of 8.8% in the WOMAC physical function score,^( 24 )^ with a standard deviation of 22, α=0.05, and 80% power, using G. Power 3.15 software, a free statistical analysis program.^( 25 )^ The results are presented as means, standard deviations, medians, quartiles, and minimum and maximum values for numerical variables. Data normality was verified using the Shapiro-Wilk normality test. Pre- and post-program measurements were compared using the paired *t* test or Wilcoxon test, depending on the data distribution.

Logistic models were adjusted to investigate the relationship between a minimum of 8.8% variation in the WOMAC score and baseline participant characteristics, EQ-5D-3L score, VAS score, sex, age, BMI, and OA degree. The results are presented as odds ratios (OR), 95% confidence intervals, and p-values.^( 26 )^

The molecules in the serum did not follow a normal distribution; therefore, logarithmic (log) transformed values were used to compare the different time points. Spearman rank correlation coefficients were used to determine the correlation between serum molecule levels and WOMAC scores. Multinomial logistic regression analysis was performed to assess the independent predictors of WOMAC scores. The relationships between the baseline levels of molecules and age, BMI, symptom duration, and baseline WOMAC score were analyzed using Spearman correlation coefficients.

The performance of variation at the molecular level in successfully discriminating patients (minimum of 8.8% in WOMAC score) was investigated by ROC curve a receiver operating characteristic (ROC) curve, and the best cutoff point was determined based on the Youden index.^( 27 )^

Analyses were performed using the SPSS statistical package for the Social Sciences version 24 for Windows (IBM; Armonk, NY, USA), with a significance level of 5%.^( 28 )^

## RESULTS

The recruitment process identified 572 individuals, 141 of whom met the eligibility criteria. After consultation with an orthopedist, 65 participants were included in the study. Of these, 11 participants discontinued the program for personal reasons, and 7 were excluded because of various conditions, including motorcycle accidents, low back pain, disabling patellofemoral pain, and failure to provide information on regular physical activity.

Table 1 presents the characteristics of the study participants. Most participants were overweight, and the mean age was 55.4 years. All participants had unilateral symptomatic OA. Knee radiographic analysis was performed by two independent orthopedic surgeons, confirming the prevalence of KL grade 2 OA.

**Table 1:** Characteristics of the participants

Sex * , n (%)		
	Female	30 (63.8)
	Male	17 (36.2)
Age [years; average (SD)]		55.4 (5.1)
	Min; Max	45;65
Weight [kg; average (SD)]		79.0 (13.1)
	Min; Max	49.5; 111.2
Body mass index [kg/m^2^; average (SD)]		
	Average (SD)	29.3 (3.5)
	Min; Max	20.5; 35.0
Symptom time (years)		
	Median (Q1; Q3)	3.0 (2.0; 4.0)
	Min; Max	0.6; 20.0
Knee alignment * , n (%)		
	Neutral	29 (61.7)
	Varus	6 (12.8)
	Valgus	12 (25.5)
Dominant side * , n (%)		
	Right	45 (95.7)
	Left	2 (4.3)
Side with complaint * , n (%)		
	Right	20 (42.6)
	Left	27 (57.4)
Radiographic KL scale * , n (%)		
	2	32 (68.1)
	3	15 (31.9)

Values expressed by average and standard deviation (SD).

* value expressed as a percentage. n=47.

KL scale=Radiographic scale in accordance with the Kellgren and Lawrence classification on the somatic side.

After 8 weeks of performing the simplified exercise program, the participants achieved a 50% reduction in the WOMAC total score, a 53% reduction in the VAS score, and an increase in the EQ-5D-3L score (p<0.001) ( Table 2 ).

**Table 2:** Measurements pre- and post-program

	Evaluation		Variation	
	Pre-program	Post-program	Contrast (Post - Pre)	p value *
EQ-5D-3L	0.623 (0.574; 0.676)	0.786 (0.744; 0.829)	0.163 (0.100; 0.227)	<0.001
VAS	2.82 (2.51; 3.16)	1.32 (1.01; 1.73)	-1.50 (-1.90; −1.10)	<0.001
TUG Osteoarthritis side	7.76 (7.43; 8.12)	7.36 (7.07; 7.67)	-0.40 (-0.57; −0.23)	<0.001
WOMAC Total score	0.38 (0.33; 0.43)	0.16 (0.13; 0.20)	-0.21 (-0.26; −0.17)	<0.001

* One-way analysis of variance (ANOVA) followed by Tukey post hoc analysis.

Values are expressed as estimates (95% confidence interval) n=47.

EQ-5D-3L: EuroQol 5-Dimension 3-Level, Quality of Life Scale. VAS: Visual Analog Scale; TUG: Timed up and Go; WOMAC: Western Ontario and McMaster Universities Osteoarthritis Index.

After the end of program, there was a significant increase in endurance on the leg press exercise, seated leg curl, and leg extension machine (p<0.001 for all) (Table 1S, Supplementary Material ). The improvement resulted in an increase in the PT and Work (W) isokinetic measures in extension exercises of the contralateral (CL) side and in flexion exercises of both sides (p<0.05 for all) as well as in isometric flexion and extension PT on both sides and concentric isokinetic F/E PT Ratio of the affected side (p<0.05 for all) ( Table 3 ).

**Table 3:** Isokinetic evaluations

Evaluation of isokinectic force		Evaluation		Variation	
		Pre-program	Post-program	Contrast (Post - Pre)	p value *
Isok extension PT (N.m/kg)					
	OA side	1.34 (1.17; 1.52)	1.45 (1.27; 1.62)	0.10 (-0.02; 0.22)	0.095
	CL side	1.46 (1.29; 1.63)	1.61 (1.44; 1.77)	0.15 (0.05; 0.25)	0.004
Isom extension PT (N.m/kg)					
	OA side	1.67 (1.45; 1.89)	1.98 (1.78; 2.19)	0.31 (0.20; 0.43)	<0.001
	CL side	1.84 (1.61; 2.07)	2.16 (1.93; 2.38)	0.32 (0.15; 0.48)	<0.001
Isom flexion PT (N.m/kg)					
	OA side	0.84 (0.73; 0.94)	1.02 (0.91; 1.12)	0.18 (0.12; 0.23)	<0.001
	CL side	0.89 (0.78; 0.99)	1.04 (0.94; 1.14)	0.15 (0.10; 0.21)	<0.001
Isok flexion PT (N.m/kg)					
	OA side	0.63 (0.53; 0.73)	0.79 (0.70; 0.89)	0.16 (0.10; 0.22)	<0.001
	CL side	0.71 (0.62; 0.81)	0.82 (0.72; 0.91)	0.11 (0.05; 0.16)	<0.001
W extension (J/kg)					
	OA side	1.11 (0.97; 1.26)	1.19 (1.05; 1.33)	0.08 (-0.03; 0.18)	0.153
	CL side	1.22 (1.08; 1.36)	1.33 (1.20; 1.46)	0.11 (0.03; 0.20)	0.007
W flexion (J/kg)					
	OA side	0.55 (0.46; 0.64)	0.69 (0.60; 0.77)	0.14 (0.08; 0.19)	<0.001
	CL side	0.61 (0.52; 0.70)	0.70 (0.62; 0.79)	0.10 (0.05; 0.14)	<0.001
C isok F/E PT ratio (%)					
	OA side	47.52 (42.41;53.24)	56.12 (50.70;62.12)	8.61 (1.98; 15.24)	0.011
	CL side	48.05 (45.06;51.23	51.19(46.90;55.87)	3.14 (-0.50; 6.78)	0.090

* One-way analysis of variance (ANOVA) followed by Tukey post hoc analysis.

Values are expressed as estimates (95% confidence interval) n=47.

Isok Extension PT: Isokinetic extension peak torque; OA side: osteoarthritic side; CL side: contralateral side; Isom Extension PT: Isometric extension peak torque; Isom Flexion PT: Isometric flexion peak torque; Isok Flexion PT: Isokinetic flexion peak torque; W Extension: Work Extension; W Flexion; Work Flexion; C Isok F/E PT Ratio: Concentric Isokinetic flexors/extensors peak torque ratio (n=47).

**Figure 1:**
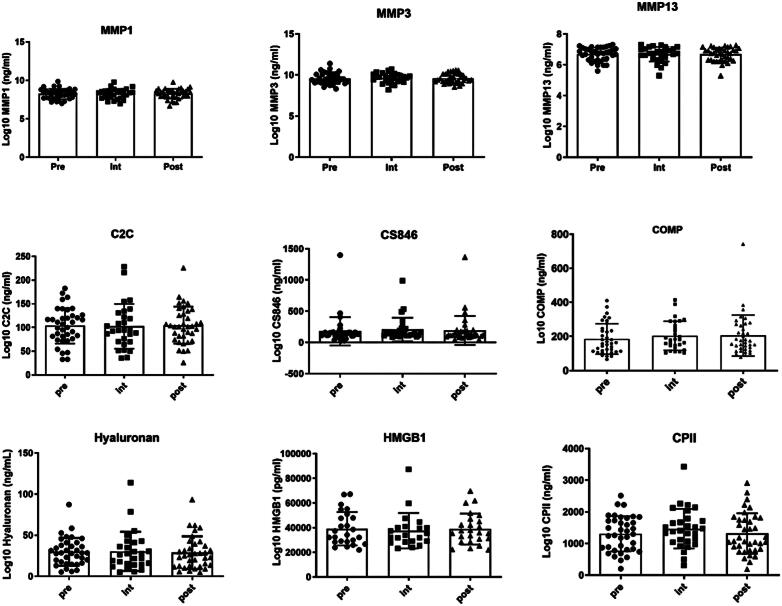
Serum levels of molecules related to cartilage degradation. Metalloproteinases (MMP) −1, −3, −13 (n=37); Type II collagen epitope (C2C; n=37), Chondroitin sulfate 846 epitope (CS846; n=37); cartilage oligomeric matrix (COMP; n=37); Hyaluronic acid (HA; n=37) and High mobility group box 1 (HMGB1; n=25); molecules related to cartilage synthesis: C-propeptide of type II procollagen (CPII; n=37); Prior to the simplified exercise program start (Pre), after 12 sessions during intervention (Int), and at the end of the program (Post)

**Figure 2:**
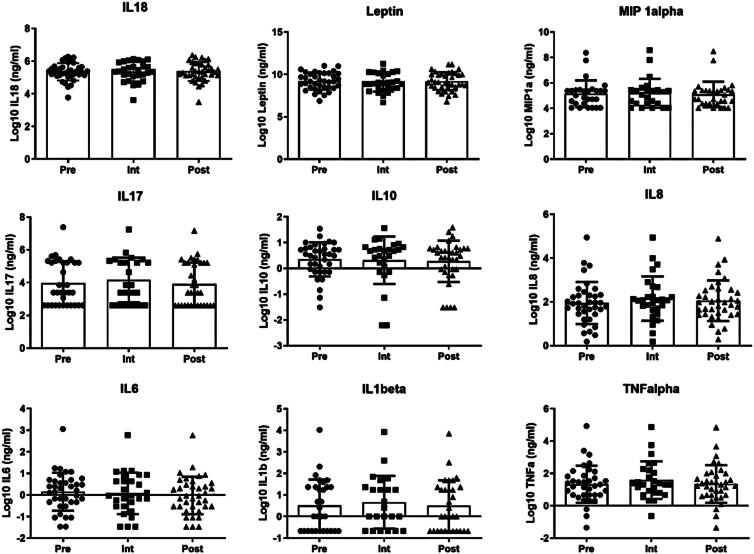
Serum levels of molecules related to inflammatory profile. Tumor Necrosis Factor (TNF)alpha; Interleukin (IL)-6; −8; −10; −1beta; −17E; −18; Macrophage Inflammatory Protein (MIP)-1alpha and Leptin (n=37). Prior to the simplified exercise program start (Pre), after 12 sessions during intervention (Int), and at the end of the program (Post)

For biomarker measurements, the analysis included 37 participants who completed the study and provided blood samples Pre, Int, and Post-program. Due to the challenges encountered in standardizing the analysis of HMGB1, evaluations were conducted on 25 samples only. We did not observe any modulation of the following molecules related to cartilage degradation: MMP-1, −3, −13, C2C, CS846, COMP, HA, HMGB1, and CPII ( Figure 1 ). There was also no change in the inflammatory profile (TNF-ALPHA; IL-6; IL-8/CXCL8; IL-10; L-1β; IL-17; CL3/MIP-1α; LEPTIN; IL-18; Figure 2 ). Relationships between basal molecule levels and age, BMI, and symptom duration (Table 2S, Supplementary Material ) were not observed.

The participants were classified as clinically successful (responders) or not (non-responders) based on an increase of at least 8.8% in the WOMAC score, which was considered the minimal clinically important difference (MCID), at the post-program evaluation compared with the baseline value. These two groups were compared in relation to the baseline levels of the analyzed molecules (Table 3S, Supplementary Material ) as well as variations in molecule levels after post-program ( Table 4 ). No association was found between the baseline molecular levels and changes in the WOMAC score or between responders and non-responders (p>0.05) in all tests.

**Table 4:** Association between modulation levels of molecules according to range after MCID program WOMAC score

Variations in molecule levels **	MCID on the WOMAC score		p value *
	No	Yes	
COMP	-24.5 (-35.0; 9.9)	11.2 (-16.1; 38.5)	0.059
	(n=7)	(n=29)	
HMGB1	-9952.1 (-20196.3; −5072.6)	2440.1 (-5263.7; 8885.8)	0.071
	(n=5)	(n=20)	
CPII	213.6 (-127.2; 395.3)	-46.2 (-320.0; 157.0)	0.192
	(n=7)	(n=29)	
C2C	-18.1 (-29.0; 11.5)	-0.7 (-16.4; 26.1)	0.192
	(n=7)	(n=29)	
CS846	-2.4 (-9.3; 27.2)	-2.3 (-17.2; 15.9)	0.503
	(n=7)	(n=26)	
HYALURONAN	-1.2 (-21.6; 0.3)	-1.6 (-7.1; 6.4)	0.505
	(n=7)	(n=28)	
MMP-1	-84.6 (-634.0; 277.9)	0.0 (-264.5; 157.0)	0.614
	(n=8)	(n=28)	
MMP-3	-130.5 (-1080.0; 796.5)	201.0 (-560.5; 1831.0)	0.513
	(n=8)	(n=28)	
MMP-13	8.2 (-16.4; 26.8)	0.0 (-16.4; 16.4)	0.78
	(n=8)	(n=28)	
TNF-ALPHA	0.11 (-0.05; 0.63)	0.00 (-0.23; 0.23)	0.251
	(n=8)	(n=28)	
IL-6	-0.03 (-0.15; 0.15)	-0.09 (-0.20; 0.05)	0.614
	(n=8)	(n=28)	
IL-8/CXCL8	1.1 (-0.9; 2.1)	0.4 (-0.7; 1.3)	0.489
	(n=8)	(n=28)	
IL-10	-0.04 (-0.18; 0.08)	0.00 (-0.22; 0.18)	0.896
	(n=8)	(n=28)	
IL-1 BETA/IL-1F2	0.00 (0.00; 0.37)	0.00 (0.00; 0.00)	0.443
	(n=8)	(n=28)	
IL-17E/IL-25	0.0 (-5.9; 6.2)	0.0 (0.0; 0.0)	0.723
	(n=8)	(n=28)	
CCL3/MIP-1 ALPHA	0.0 (0.0; 9.9)	0.0 (-6.6; 6.8)	0.537
	(n=8)	(n=28)	
LEPTIN	339.5 (-508.0; 1743.5)	-744.0 (-2228.0; 2372.0)	0.251
	(n=8)	(n=28)	
IL-18	0.0 (-18.6; 95.4)	-1.1 (-12.7; 41.6)	0.837
	(n=8)	(n=28)	
AGGRECAN	0.0 (0.0; 0.0)	0.0 (-4.0; 0.0)	0.641
	(n=8)	(n=28)	

* Nonparametric Mann–Whitney test; data are expressed as medians (first and third quartiles).

** Variations in molecule levels between baseline and post-program.

MCID: minimal clinically important difference, increase of at least 8.8% in the Western Ontario and McMaster Universities Osteoarthritis Index. No: Participants who did not achieve an increase of at least 8.8% in WOMAC. Yes: Participants who achieved an increase of at least 8.8% in WOMAC.

We also determined a cutoff value for the 19 molecules to identify participants who successfully achieved clinical outcomes based on the post-program WOMAC score. We found the observed values of the area under the ROC curves for variations in molecules in relation to succession and verified that these measures of variation did not perform well in determining clinical success (Table 4S, Supplementary Material ).

## DISCUSSION

The simplified exercise program that focused on strengthening exercises of three lower limb muscle groups is effective in the management of patients with mild to moderate knee OA as evidenced by an improvement in the WOMAC score, EQ-5D-3L scale, VAS, TUG, endurance exercises, and isokinetic parameters. This simplified exercise program can be used by various individuals with OA regardless of age, sex, degree of OA, BMI, and/or pain. Exercise therapy is an effective intervention for reducing joint pain and improving physical function in patients with knee OA and is recommended as the primary non-drug intervention by international guidelines.^( 7 )^ Patients’ demographic data in this study were similar to those of populations analyzed in other studies using different rehabilitation protocols.^( 29 , 30 )^ The majority of cases in this study involved the left side, with a prevalence of 57.4%. Conversely, research analyzing the impact of home-based exercises indicated a higher frequency of involvement of the right side.^( 31 )^

Bokaeian et al.^( 32 )^ evaluated the correlation between increased quadricep muscle strength and improved pain and function in patients with knee OA. Twenty-four patients participated in an 8-week treatment protocol that included traditional physical therapy and strength training, with three sessions per week. This study reported the absence of a significant correlation among quadriceps muscle strength, pain, and functional activity caused by strength-training protocol therapies in patients with knee OA. However, we believe that this study had an extremely small sample size. In contrast, our sample demonstrated an improvement in all objective and subjective parameters, in accordance with the results of other studies.^( 33 - 35 )^

OA is associated with the deterioration of health-related quality of life.^( 36 )^ The study classified OA as the third leading cause of loss of quality of life among 11 chronic health conditions.^( 37 )^ Through the EQ-5D-3L, we demonstrated, we demonstrated that a simplified exercise protocol produces results similar to those from another study that involved a 90-minute exercise program twice a week for 12 weeks.^( 38 )^ Another study showed that individuals with OA and knee pain have lower EQ-5D-3L scores, which negatively impacts their quality of life.^( 39 )^ In contrast, our participants reported median scores on the EQ-5D-3L scale before the program, which may have contributed to a significant increase in their quality of life.

Our results also demonstrated a significant reduction in the time required to complete the TUG test. In contrast, a previous study evaluating a 2-day educational program with self-care guidelines and home exercises for patients with mild-to-moderate knee OA did not show changes in TUG after 1 year.^( 40 )^ Another study evaluating resistance exercises of the quadriceps performed twice a week for 8 weeks showed a significant improvement in the TUG test at the end of treatment.^( 41 )^

An imbalance analysis of the muscular forces on the knee is important for injury prevention. In a joint, one of the strategies used to obtain this result is the calculation of the conventional ratio, which is obtained by dividing the concentric flexor PT by the concentric extensor PT of the same member.^( 42 , 43 )^ The value of 60% from this equation has been suggested as ideal for joint balance in isokinetic evaluation at a speed of 60°.s^-1^, and subjects who present this value have a reduced risk of injury to the knee joint.^( 20 , 43 , 44 )^ The concentric F/E PT ratio showed that this value was not reached by our patients, although we observed muscle strength improvement in the lower limb after the simplified exercise program. An important advantage of this study was the strict exclusion of participants with OA from the anterior knee compartment as we believe that a rehabilitation program for these patients has specific components that differentiate it from those involving the medial and lateral compartments.

Studies^( 4 , 6 )^ have also investigated the correlation between biomarker levels and OA progression, and we observed that strong candidates for this correlation were COMP, HA, and MMP3. A meta-analysis^( 45 )^ suggested that serum levels of COMP and urinary CTX-II may distinguish patients with OA from controls, and that COMP is the most effective biomarker for predicting OA progression.^( 46 , 47 )^

However, few studies have analyzed the effects of these biomarkers on the clinical evolution of patients with OA after conservative treatment. A recent randomized clinical trial investigated the effects of incorporating photobiomodulation (PBM) into a physical exercise program on serum inflammatory biomarkers.^( 5 )^ The protocol involved 8 weeks of exercise (twice a week), including warm-up, six strength exercises, and stretching, with or without PBM application (808 nm, 56 J per knee) after each session. Corroborating our findings, their protocol revealed a significant improvement in functional capacity (assessed using the WOMAC) in the groups that performed exercises regardless of PBM application. In contrast, the group that underwent exercise combined with active PBM demonstrated a significant increase only in IL-10 levels and no difference in CTX-II levels compared to the controls. Although this result is promising, the study had 13 participants per group and did not include a group that received PBM alone without exercise to isolate the effects.

A study with 1,335 participants and a 5-year follow-up period demonstrated an association between urinary CTX-II levels and serum COMP levels with disease progression.^( 48 )^ This finding has also been corroborated by other studies, indicating that individuals with OA have higher serum COMP levels than those without the disease and suggesting that COMP may serve as an indicator of structural damage to the cartilage.^( 45 , 49 , 50 )^ Notably, serum COMP levels increase immediately after cyclic mechanical loading, such as in moderate walking^( 48 )^ and running^( 9 , 10 )^ activities. One study suggested that an exercise program contributes to the maintenance of the synthesis and degradation of articular cartilage components in individuals with OA.^( 11 )^ The authors demonstrated that serum COMP levels increased after 24 weeks of exercise, whereas there was a reduction in urine C2C levels after 12 weeks. More importantly, patients in that study had a KL 0/1 OA classification; in our study, the majority had a KL 2 OA classification (68%; 32 participants). The authors mentioned that a limitation of their study was the lack of control in the execution of the exercises because after 12 face-to-face weekly sessions, the patients were encouraged to continue the exercises at home, and exercise execution was not controlled. In our study, the patients were accompanied by a physical therapist throughout the program that was conducted thrice weekly.

An important advantage of our study was the rigorous control used to exclude participants with OA of the anterior knee. We believe that the simplified exercise program for these patients has unique components that distinguish them from those involving medial and lateral compartments. However, some limitations should be considered when interpreting the results of this study. The lack of a Control Group is the primary limitation, making it challenging to interpret the relationship between biomarker levels and disease.

It would be intriguing to conduct a randomized study to compare the results of this simplified exercise program with those of another conventional program, or with no rehabilitation; however, some challenges must be considered. First, it was not possible to blind the participants, which could have affected the objectivity of the results. Additionally, conducting such studies requires a larger sample size, leading to higher costs. Moreover, ethical concerns may arise when considering the use of placebo or no treatment. Lastly, using historical controls may not be suitable because of differences in population samples and the inability to control for various biases, particularly selection bias.

## CONCLUSION

The simplified exercise program to strengthen three muscle groups in the lower limbs of patients with mild-to-moderate knee osteoarthritis was effective in improving pain, functionality, muscle strength, and quality of life. However, biomarkers do not provide evidence for monitoring physical improvement after the program.
