# Supplementary material for: Effect of simplified exercise program on quality of life, biomarkers, pain and muscle strength of individuals with knee osteoarthritis
Source: Einstein (Sao Paulo). 2025 Nov 13;23:eAO1192. doi: 10.31744/einstein_journal/2025AO1192 (PMC12671636; doi:10.31744/einstein_journal/2025AO1192)
Supplement: Supplementary file 1 [file 2317-6385-eins-23-eAO1192-suppl1.pdf]

SUPPLEMENTARY MATERIAL

Effect of simplified exercise program on quality of life, biomarkers, pain and muscle strength of individuals with knee osteoarthritis

Rosana Ravagnani Campedelli, Mariana Rosada de Souza Jardim, Eliane Antonioli, Felipe Bruno Dias de Oliveira, Sudha Agarwal, Mario Ferretti

DOI: 10.31744/einstein_journal/2025AO1192

Table 1S. Endurance exercises at one repetition maximum test

Endurance exercises (1RM)	Evaluation		
	Pre-program	Post-program	Mean (95%CI)
Leg press (kg)	97.4 (85.4; 109.3)	143.0 (131.2; 154.9)	<0.001
Seated leg curl machine (kg)	34.7 (31.0; 38.8)	44.2 (39.7; 49.2)	<0.001
Leg extension machine (kg)	38.2 (33.5; 43.6)	47.0 (41.5; 53.1)	<0.001

1RM: One-Repetition Maximum.Values expressed through estimated values (95%CI). (n=47).

Table 2S. Coefficients of correlation between baseline levels of molecules and age, body mass index, and duration of symptoms in participants with knee osteoarthritis

Baseline levels of molecules	Patient characteristics		
	Age	Body mass index	Symptoms time
COMP (n=36)	0.162 (p=0.345)	0.254 (p=0.134)	-0.046 (p=0.788)
HMGB1 (n=25)	-0.338 (p=0.098)	0.234 (p=0.261)	0.150 (p=0.473)
CPII (n=36)	0.051 (p=0.768)	-0.296 (p=0.080)	0.133 (p=0.441)
C2C (n=36)	-0.011 (p=0.951)	-0.184 (p=0.284)	-0.140 (p=0.417)
CS846 (n=33)	-0.127 (p=0.481)	0.228 (p=0.201)	0.093 (p=0.606)
HYALURONAN (n=35)	0.385 (p=0.023)	0.298 (p=0.082)	0.246 (p=0.155)
MMP-1 (n=36)	0.038 (p=0.826)	0.331 (p=0.048)	0.149 (p=0.385)
MMP-3 (n=36)	0.038 (p=0.825)	0.037 (p=0.832)	-0.197 (p=0.250)
MMP-13 (n=36)	0.156 (p=0.364)	0.145 (p=0.399)	-0.159 (p=0.353)
TNF-ALPHA (n=36)	0.032 (p=0.854)	0.195 (p=0.255)	-0.200 (p=0.242)
IL-6 (n=36)	0.026 (p=0.881)	-0.070 (p=0.684)	-0.321 (p=0.056)
IL-8/CXCL8 (n=36)	0.075 (p=0.665)	0.125 (p=0.468)	-0.121 (p=0.482)
IL-10 (n=36)	-0.031 (p=0.857)	0.139 (p=0.418)	0.040 (p=0.816)
IL-1 BETA/IL-1F2 (n=36)	-0.057 (p=0.742)	0.112 (p=0.517)	0.014 (p=0.933)
IL-17E/IL-25 (n=36)	-0.008 (p=0.963)	0.365 (p=0.029)	0.064 (p=0.711)
CCL3/MIP-1 ALPHA (n=36)	-0.163 (p=0.342)	0.241 (p=0.156)	0.201 (p=0.239)
LEPTIN (n=36)	-0.058 (p=0.735)	0.171 (p=0.318)	0.004 (p=0.984)
IL-18 (n=36)	0.077 (p=0.656)	-0.055 (p=0.748)	-0.142 (p=0.408)
AGGRECAN (n=36)	-0.045 (p=0.793)	-0.005 (p=0.975)	-0.340 (p=0.042)

Data expressed by Spearman's correlation coefficient (p-value).

Table 3S. Association between baseline molecules levels and MCID classification by WOMAC

Baseline levels of molecules	MCID on the WOMAC score		p value*
	No	Yes	
COMP	226.1 (113.2; 336.1) (n=7)	158.3 (116.4; 231.8) (n=29)	0.531
HMGB1	51048.0 (41221.6; 58907.0) (n=5)	32265.2 (27708.7; 44662.7) (n=20)	0.097
CPII	808.3 (589.3; 1261.0) (n=7)	1435.0 (932.4; 1732.0) (n=29)	0.065
C2C	116.7 (81.7; 126.6) (n=7)	107.4 (81.7; 124.9) (n=29)	0.938
CS846	137.1 (90.7; 154.5) (n=7)	133.7 (107.2; 160.3) (n=26)	0.503
HYALURONAN	31.3 (15.3; 45.6) (n=7)	28.3 (18.8; 39.3) (n=28)	0.702
MMP-1	2129.0 (1084.6; 3404.5) (n=8)	2074.0 (1184.5; 2917.0) (n=28)	0.837
MMP-3	9605.0 (5337.0; 15138.5) (n=8)	6764.0 (4758.5; 10978.5) (n=28)	0.339
MMP-13	418.0 (352.4; 475.8) (n=8)	377.0 (332.0; 418.0) (n=28)	0.267
TNF-ALPHA	3.59 (2.69; 6.68) (n=8)	3.46 (2.79; 4.89) (n=28)	0.867
IL-6	1.20 (1.11; 1.30) (n=8)	1.20 (0.90; 1.76) (n=28)	0.78
IL-8/CXCL8	8.4 (5.4; 11.6) (n=8)	6.0 (3.2; 8.9) (n=28)	0.302
IL-10	1.49 (1.16; 2.10) (n=8)	1.57 (1.32; 1.92) (n=28)	0.837
IL-1 BETA/IL-1F2	2.69 (2.32; 4.02) (n=8)	2.69 (2.69; 3.25) (n=28)	0.837
IL-17E/IL-25	83.8 (72.0; 103.3) (n=8)	72.0 (72.0; 90.0) (n=28)	0.339
CCL3/MIP-1 ALPHA	83.9 (77.2; 98.4) (n=8)	83.9 (77.2; 106.1) (n=28)	0.489
LEPTIN	4018.0 (2343.0; 22863.5) (n=8)	12159.5 (6615.0; 24950.0) (n=28)	0.107
IL-18	247.9 (181.6; 459.5) (n=8)	197.9 (153.0; 243.3) (n=28)	0.099
AGGRECAN	95.7 (95.7; 100.2) (n=8)	95.7 (95.7; 118.0) (n=28)	0.302

* Nonparametric Mann–Whitney test; data expressed as medians (first quartile; third quartile). MCID: minimal clinically important difference, increase of at least 8.8% in the Western Ontario and McMaster Universities Osteoarthritis Index.

Table 4S. Measures of performance of the ROC curves for the variations in the levels of the molecules in relation to the success (MCID in the variation of the WOMAC score)

Molecule	ASC (95%CI)	p value	Cutoff*	Sensitivity	Specitivity
COMP	0.734 (0.535; 0.933)	0.058	-10 500	0.724	0.714
HMGB1	0.770 (0.493; 1.000)	0.067	-4 992 300	0.750	0.800
CPII	0.665 (0.463; 0.867)	0.181	111 891	0.690	0.714
C2C	0.665 (0.459; 0.871)	0.181	-14 120	0.724	0.714
CS846	0.588 (0.377; 0.799)	0.481	-15 150	0.308	1 000
HYALURONAN	0.587 (0.333; 0.840)	0.483	-11 900	0.893	0.429
MMP-1	0.563 (0.309; 0.816)	0.594	-515 000	0.857	0.375
MMP-3	0.578 (0.361; 0.795)	0.505	1 041 500	0.321	0.875
MMP-13	0.533 (0.292; 0.775)	0.775	16 315	0.714	0.500
TNF-ALPHA	0.641 (0.416; 0.865)	0.231	0.445	0.964	0.375
IL-6	0.560 (0.332; 0.788)	0.607	-0.070	0.536	0.625
IL-8/CXCL8	0.585 (0.352; 0.817)	0.470	0.710	0.643	0.625
IL-10	0.520 (0.303; 0.738)	0.864	0.115	0.321	0.875
IL-1 BETA/IL-1F2	0.594 (0.357; 0.831)	0.424	0.185	0.786	0.375
IL-17E/IL-25	0.545 (0.298; 0.791)	0.704	12 070	0.929	0.250
CCL3/MIP-1 ALPHA	0.576 (0.355; 0.796)	0.518	-1 700	0.321	0.875
LEPTIN	0.638 (0.455; 0.822)	0.238	-1 226 000	0.429	1 000
IL-18	0.527 (0.269; 0.784)	0.819	83 170	0.893	0.375
AGGRECAN	0.556 (0.327; 0.785)	0.634	-4 045	0.250	0.875

* Values of variation in the levels of molecules were obtained based on the Youden index, from which values of sensitivity and specificity were obtained.
MCID: minimal clinically important difference; WOMAC: Western Ontario and McMaster Universities Osteoarthritis Index; ASC: area under the ROC curve; 95% CI: 95% confidence interval.
